# Seasonality and maximum wind gusts correlate with microplastic concentrations in a freshwater stream system

**DOI:** 10.1007/s10661-026-15985-9

**Published:** 2026-10-09

**Authors:** Chloe Mattie, Isabella Ashman, Megan Fork, T. Elliott Arnold

**Affiliations:** 1 Department of Earth and Space Sciences, West Chester University, West Chester, PA USA; 2 Department of Biology, West Chester University, West Chester, PA USA

**Keywords:** Microplastics, Stepwise, Environmental, Spectroscopy, Freshwater

## Abstract

We evaluate how meteorological and environmental variables relate to stream microplastic concentrations using regression-based models. Forty-nine water column samples were collected bimonthly over an annual cycle (September 2024–September 2025) from a stream in southeastern Pennsylvania, USA. Microplastic particles were isolated and quantified by light microscopy, and polymer types were characterized using scanning electron microscopy with energy-dispersive X-ray spectroscopy (SEM–EDS) and Fourier transform infrared spectroscopy (FTIR). Results from three separate multiple linear regression models (stepwise selection, LASSO, and elastic net) demonstrate high variability in microplastic counts, including pronounced seasonal variability (mean = 7.6 particles L⁻^1^, *n* = 49, 1σ = 4.9 particles L⁻^1^). All regression approaches identified colder temperatures and stronger wind gusts as important predictors of elevated microplastic concentrations. Repeated tenfold cross-validation indicated that models incorporating these variables explained approximately 80% of the variability in microplastic concentrations within this relatively small dataset (*n* = 49), highlighting their importance as the dominant environmental controls on microplastic abundance in Plum Run. When scaled by riparian area, the wind-sensitive component of the stream plastic flux is consistent with physically plausible atmospheric deposition rates. These results provide an indirect inference that environmental factors enhance transport of microplastics into waterways. Qualitative data from EDS and FTIR spectra indicate that polyester fibers comprise the majority of microplastic pollution in Plum Run. From this, we infer that increased laundering and shedding of synthetic clothing during winter may be a primary driver of higher pollution in winter months.

**Supplementary Information:**

The online version contains supplementary material available at https://doi.org/10.1007/s10661-026-15985-9.

## Introduction

The degradation of freshwater systems is closely intertwined with an exponential increase in human populations and the concomitant deposition of pollutants in aquatic systems (Akhtar et al., 2021; Bhardwaj et al., 2024; Sunny et al., 2025). Of these pollutants, plastic and microplastic pollution represents a major contributor to the observed deterioration in marine (Agbekpornu & Kevudo, 2023) and freshwater systems (Bhardwaj et al., 2024). Plastics are manufactured and used extensively in industrial and residential settings, and as such, plastic pollution is produced from a range of anthropogenic activities. Numerous studies document the pervasiveness of plastic polymers in aquatic systems (Gall & Thompson, 2015; Mishra et al., 2022) and link this pollution to a range of terrestrial sources (Kallenbach et al., 2022).

Once introduced to aquatic environments, plastics undergo physical, chemical, and biotic degradation that fragments larger items into microplastics (particles < 5 mm), while microplastics may also enter streams directly as primary particles from manufacturing, personal care products, or industrial abrasion (Sunny et al., 2025). Despite growing recognition of the risks plastics pose, temporal variability in stream microplastic concentrations remains poorly constrained. Studies relying on periodic grab sampling may substantially underestimate or mischaracterize concentration dynamics, as concentrations in a single stream have been shown to vary by more than an order of magnitude over periods of days (Kukkola et al., 2024; Witzig et al., 2024). The mechanisms driving this variability, including hydrological discharge, atmospheric deposition, and seasonal changes in anthropogenic activity, remain incompletely understood, particularly in temperate, nonmonsoonal climates where precipitation may not be the dominant control (Montecinos et al., 2024; Soininen et al., 2024; Wright et al., 2020).

**Fig. 1:**
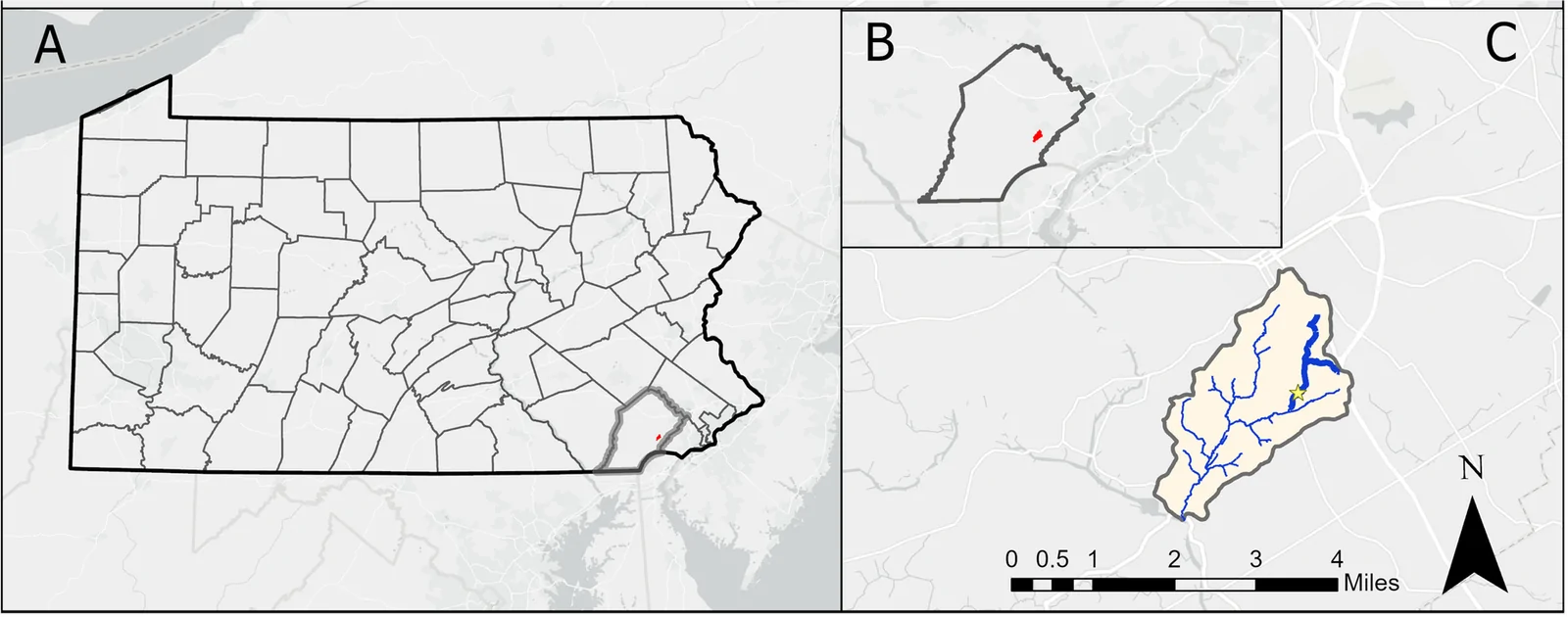
Composite map showing **A** the location of Chester County within Pennsylvania, USA, **B** the lower Brandywine Watershed within Chester County (in red), and **C** an enlarged map of the watershed with Plum Run bolded in blue and our sampling location indicated by a star

**Fig. 2:**
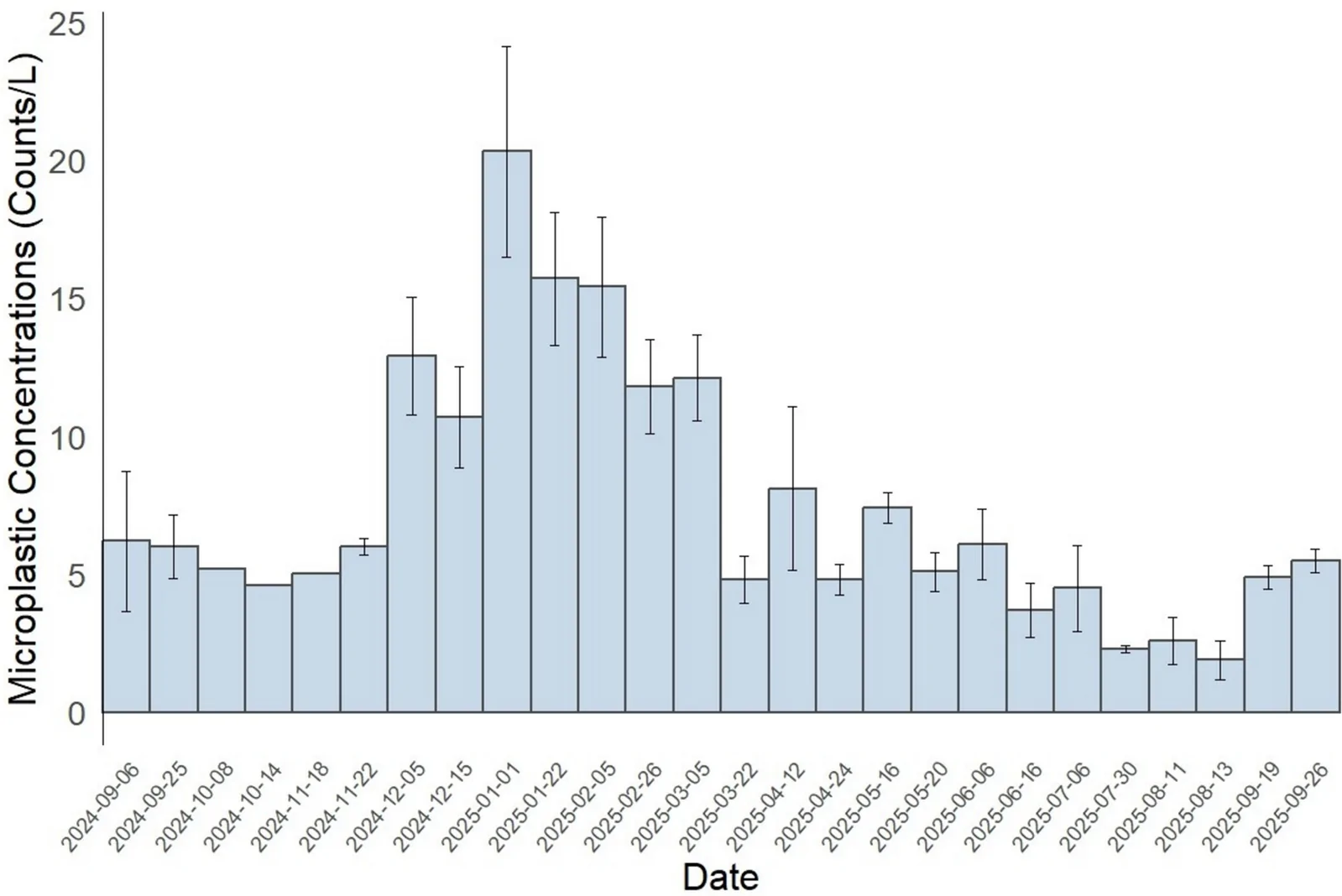
Mean daily microplastic counts from each Plum Run sampling trip, with 1σ error bars

Against this backdrop, our study has three primary aims: (1) to identify the environmental and meteorological variables most strongly associated with temporal variability in microplastic concentrations in Plum Run, a Piedmont stream in southeastern Pennsylvania, USA (Fig. 1); (2) to characterize the polymer composition and morphology of microplastics recovered from the stream to help constrain likely source categories; and (3) to evaluate whether the resulting environmental associations are consistent with wind-driven atmospheric (aeolian) delivery of microplastics to the stream corridor, a pathway that has received comparatively little attention relative to hydrologically driven transport (Bullard et al., 2021; Wright et al., 2020). To address these aims, we collected 49 water column samples from Plum Run across an annual cycle and used multiple linear regression approaches (stepwise selection, LASSO, elastic net) to identify which environmental variables best explain microplastic concentrations across the sampling period.

## Materials and methods

### Study area

Plum Run is a second-order Piedmont stream approximately 19.3 km in length, with a mean annual discharge of 0.03 m^3^ s⁻^1^, located in West Chester, Pennsylvania, USA (Fig. 1). The stream drains a predominantly urbanized watershed and flows into the East Branch of the Brandywine Creek, which is part of the Christina River basin and ultimately discharges into the Delaware Bay. The East Branch Brandywine Creek Watershed covers an area of 316 km^2^ and has undergone extensive urbanization and loss of forested and agricultural land use areas since the 1990 s (Coutu & Vega, 2007) .

West Chester is one of the most densely populated municipalities in Chester County, with a population of approximately 18,000 residents. Residential land use comprises roughly 50% of the Plum Run watershed, with additional contributions from commercial and industrial-related land use zones (Ryan, 2022). No permitted combined sewer overflow discharge points or wastewater plant outfalls are documented within the Plum Run watershed; however, municipal separate storm sewer system outfalls discharge to the stream throughout its urbanized reaches. The watershed contains extensive impervious surfaces and stormwater conveyance infrastructure, which enhance surface runoff and promote rapid hydrologic response during precipitation events. These characteristics could enhance the mobilization and downstream transport of particulate pollution derived from urban sources (Coutu & Vega, 2007).

West Chester lies at the boundary between humid continental and humid subtropical climate zones, with a mean annual air temperature of 13.15 °C and average annual precipitation of approximately 120 cm (Center for Environmental Monitoring and Analysis, 2004). Precipitation is relatively evenly distributed throughout the year, although high-intensity rainfall events associated with convective storms are common during summer months and can generate short-lived but elevated discharge conditions. Such hydrologic variability could influence both the transport efficiency and temporal variability of plastic concentrations in the stream.

### Microplastic sampling

From September 2024 to September 2025, we conducted bimonthly water column sampling at 39.935°N, −75.599°W within the mainstem of Plum Run. Water samples were collected using two replicate 5-L bottles per event on most dates; however, only single samples were available for three dates (10/8/2024, 10/14/2024, and 11/18/2024) due to sample loss during processing. Each water sample was filled from the center of the stream’s water column using separate 2-L beakers. After collection, each bottle was capped immediately with a precombusted aluminum foil-lined lid, labeled, transported to the laboratory in a covered cooler, and refrigerated at 4 °C until processing within 48 h of collection. Replicate samples were processed independently, with per-event means calculated from these replicates. Water was passed through a vacuum filtration apparatus, and plastics were collected on the surface of a 47-mm fritted glass support base (10 µm) with a metal mesh (50 µm) placed on top. The contents on the surface of the frit and mesh were placed in an oven at 50 °C to evaporate residual water overnight.

To isolate microplastics from the organic matrix, organic matter was oxidized via 30% H_2_0_2_ and Fenton’s reagent. Approximately 2 mL of each solution was added to the Petri dish with the mesh, and the reaction was monitored at room temperature using a temperature probe until completion. Although the reaction was initiated at room temperature, its exothermic nature produced brief temperature increases to approximately 70–80 °C before returning toward ambient conditions. Similar transient temperature increases have been reported during wet peroxide oxidation, with complete or nearly complete recovery of synthetic fibers, fragments, and shavings despite reaction temperatures in excess of 80 °C (Munno et al., 2018). Wet peroxide oxidation was repeated, at the 2-mL volumes, until the organic matter was completely oxidized. Oxidized samples were dried overnight in an oven at 50 °C. Many samples exhibited a precipitate coating on the mesh and glassware. To dissolve the precipitate, 2 mL of 0.1N HCl was added to the affected samples, and the samples were once again dried in the oven at 50 °C.

After samples were oven-dried, they were counted via light microscopy (SI Fig. 1). Particles were counted using a systematic grid-based approach to prevent duplicate counting. A grid was marked on the underside of the glass Petri dish prior to collection, with each grid cell approximately corresponding to the microscope field of view. The mesh was examined sequentially from left to right and top to bottom, with each field of view counted once to ensure complete coverage without recounting particles. Following examination of the mesh, it was carefully removed and the underlying interior surface of the Petri dish was examined using the same systematic approach to account for particles < 50 µm that may have passed through the mesh. Particles were identified as microplastics under the light microscope based on the following criteria: (1) size between 10 µm and 5 mm, (2) uniform color or coloration inconsistent with natural organic material, (3) no observable cellular or fibrous plant structure, and (4) characteristic synthetic appearance (smooth surface and uniform diameter for fibers) (De Frond et al., 2022; Norén, 2007). Seasonal differences in average microplastic concentrations per sampling event were evaluated by comparing winter (November–March) and summer (May–September) sampling periods using Welch’s unequal-variances *t* test, and results are reported as the difference in means with a 95% confidence interval.

Plastics were classified into morphological categories based on visual appearance under the light microscope. Fibers were defined as elongated, flexible particles with a length-to-width ratio > 5 that could be bent without fracturing. Films were identified as thin, flat, transparent or semitransparent sheets. Fragments were irregular, rigid particles with rounded or angular edges, whereas pellets/spherules were classified as rounded to near-spherical particles. If clusters of microplastics were found within a sample, they were separated via sonication and forceps and then counted individually.

### SEM and FTIR analyses

SEM/EDS analysis was used as a morphological screening tool to identify the size and shape of microplastics and to provide preliminary polymer class assignments based on elemental ratios. In this study, EDS was used as an elemental technique that cannot provide definitive molecular identification and cannot distinguish among carbon-rich polymers with overlapping elemental compositions. Particle dimensions were measured from a subset of 75 SEM micrographs using AZtec software. Fiber length was measured along the longest visible axis, whereas fiber width was measured perpendicular to the longitudinal axis at representative locations along the particles. SEM imaging was conducted at 5.0 kV to prevent potential damage to the particles and EDS analyses at 20.0 kV. In addition to our samples, a suite of known polymer class standards was analyzed to determine elemental ratios for each polymer class (SI Fig. 2). The elemental ratios were then used as reference points to classify microplastics identified in samples.

Due to the semiquantitative diagnostic nature of EDS, a subset of 30 microplastic particles previously analyzed through SEM–EDS were analyzed using a Thermo Fisher iS5 Fourier Transform Infrared Spectrometer (FTIR) coupled with a diamond attenuated total reflection (ATR) crystal. The ATR-FTIR instrument measures infrared absorbance relative to a background spectrum. The resulting absorbance values are converted by the instrument software to percent transmittance using the relationship $\mathrm{\%}T=100\times {10}^{-A}$, where A is absorbance. Each particle was assigned a polymer identity by manually confirming the peak correspondence for the diagnostic absorption bands at relevant wavenumbers with our known polymer class standards (Supplemental Table 1). FTIR-based classifications were then evaluated against initial SEM–EDS identifications to verify polymer assignments and assess the consistency of the two analytical methods.

### Quality assurance and quality control

Prior to sample collection, all glassware was immersed into an acid bath for 24 h, triple rinsed with deionized water, and combusted at 450 °C for 5 h. Glassware was stored covered in combusted aluminum foil. In the field, all water samples were sealed immediately after collection and refrigerated by field personnel who wore 100% cotton clothing during sampling. Microplastic samples were processed in the lab, within 30 days of collection, in a laminar flow hood equipped with a HEPA filter to minimize airborne contamination from synthetic fibers and particulates. Recovery tests (*n* = 3) were conducted by spiking procedural blanks with 30 polyester fibers ranging from 0.25 to 5 mm in length. Spiked samples were vacuum filtered and subjected to oxidation using 30% H_2_O_2_ and Fenton’s reagent following the same procedures applied to environmental samples prior to drying and analysis. Recovery tests yielded a mean efficiency of 98% (range = 95–100%).

Throughout the sampling period, one combined field and method blank (*n* = 13) was collected monthly, corresponding to one blank for every two environmental samples, and each blank was processed in a manner identical to stream water samples. The 5-L media bottles used for blank collection were taken into the field, opened and resealed, and transported back to the laboratory alongside the samples. Media bottles were then filled with 5 L of deionized water to transfer their contents onto the vacuum filter. Because individual blanks could not be uniquely associated with contamination experienced by individual samples, blank values were averaged within each season to estimate the background contamination during the corresponding sampling and processing period. Seasonal mean blank values were subtracted from total particle counts of samples collected during the corresponding season. Mean blank abundances were highest during the winter months (December–March), averaging 12 particles per blank (range: 6–17 particles). Summer blanks averaged 6 particles (range 3–11 particles), whereas spring and fall blanks each averaged 8 particles per blank (range 6–15 particles). All microplastic particles found in blank samples were of fibrous morphology.

### Environmental variables and stepwise selection model

Historical meteorological data was taken from the closest monitoring station located in West Chester, PA (39.94°N, 75.55°W) (Center for Environmental Monitoring and Analysis, 2004), located approximately 8 km from the sampling location. Temperature and wind speed were averaged across a 24-h period prior to sample collection, while precipitation was totaled over this period. In addition, the highest wind gust in the 24-h period was recorded. Discharge was calculated by developing an exponential relationship between Acoustic Doppler Current Profiler measurements (m^3^ sec^−1^) and long-term stream gauge data (m) recorded on a Mayfly station in Plum Run (Stroud Water Research Center, 2017; https://monitormywatershed.org/sites/BCPR8S/) and the average discharge for the 24-h period prior to sample collection used as a predictor.

Historical meteorological data and microplastic counts were incorporated into a stepwise regression framework in R using the MASS package, with model performance evaluated using the Akaike information criterion (AIC) (Venables & Ripley, 2002). The following environmental parameters were included as predictors in the model: total precipitation, average discharge, average wind speed, maximum wind gust, wind direction, and average temperature. To assess the combined effects of these variables, we added a set of interacting predictors defined as follows:

1 $$X_{3}=X_{1}X_{2}$$

where *X*_3_ is the constructed interacting variable, which is a combination of predictor *X*_1_ and another predictor *X*_2_. The following interacting variables were included in the model: total precipitation × avg wind speed, total precipitation × maximum wind gust, total precipitation × average temperature, total precipitation × discharge, average wind speed × maximum wind gust, average wind speed × average temperature, average wind speed × discharge, maximum wind gust × average temperature, maximum wind gust × discharge, and average temperature × discharge.

The stepwise regression model identifies which environmental variables best explain microplastic concentrations in the stream across the entire sampling period. We applied a stepwise selection in both directions, meaning the model iteratively adds and removes independent variables until only the most influential predictors remain. An equation is made from the model to predict microplastic concentrations based on the average environmental conditions recorded across the 24-h period prior to collection.

We assessed the effects of multicollinearity in our model using the alias() and the vif() functions in R (R Core Team, 2025). The alias function indicates if any variables are perfect linear combinations, while the vif function quantifies how much of the variance of a regression coefficient is inflated due to multicollinearity. Multicollinearity occurs when two or more (independent) predictor variables in a regression model are highly correlated. When this occurs, it may artificially inflate the predictive power of these variables on the predictand. To reduce redundancy and improve model stability, any combination of variables flagged via the alias function and those with vif > 10 was removed from the final model.

Because environmental predictors of microplastic concentrations are often intercorrelated, we complement stepwise selection with penalized regression approaches. We implemented both least absolute shrinkage and selection operator (LASSO) and elastic net regression models on the same dataset in R (R Core Team, 2025). LASSO regression was used to identify the most parsimonious set of predictors by shrinking weaker coefficients to zero, whereas elastic net regression was applied to balance variable selection with grouped retention of correlated predictors. Penalized regression models were fitted using the glmnet package in R (Friedman et al., 2010), with optimal penalty parameters (*λ* and *α* for elastic net) selected via tenfold cross-validation (100 repetitions) implemented in the cv.glmnet function. We similarly added cross-validation around a stepwise model to test its predictive performance with a k-fold cross-validation (*k* = 10, 100 repetitions) using the caret package in R (Kuhn, 2008). With this approach, our dataset was randomly divided into 10 equal parts (folds), and our models were trained on 9 folds (the training set) and tested on the remaining fold (the validation set). The cross-validation process is repeated 100 times with each fold serving as the validation set once. Separately, the stepwise regression models, implemented with MASS and caret (Venables & Ripley, 2002), performed variable selection within each resample to prevent information from the validation data influencing model training, though model structure could vary among folds due to the heuristic nature of AIC-based selection. The root mean squared error (RMSE) from each cross-validation iteration for all three models was averaged and used as a robust estimate of model accuracy, with lower RMSE values indicating better model performance.

## Results

### Microplastic analyses

Particle lengths in the SEM–EDS subset ranged from approximately 45 to 1150 μm (median 235 μm), and widths ranged from approximately 7 to 25 μm (median 18 μm). The average aspect ratio (maximum length/average width) (Auyang et al., 2025) of the fibrous microplastic particles was 14 (range = 6–85). After subtracting monthly averaged blank totals, we counted 1856 microplastic particles across all 49 samples. Microplastic abundances across the sampling period ranged from 1.2 to 23.0 particles L^−1^, with a mean of 7.6 particles L^−1^ (*n* = 49, 1σ = 4.9 particles L^−1^). Microplastic counts were lower (556 total particles) in the spring and summer months between May and September, when average temperatures were above 13°C. In the fall and winter months, when average temperatures were below 13 °C, microplastic counts were higher at 1122 total particles (Fig. 2). Mean microplastic concentrations were approximately 4.7 particles L⁻^1^ (± 1.7 particles) during the warm season (May–September) compared to 10.6 particles L⁻^1^ (± 4.8) during the cool season (November–March), an approximately twofold difference.

Fibrous morphologies dominate our microplastic assemblages. Microplastics analyzed through SEM–EDS were classified as fibers (96%), films (3%), and fragments (1%). There was no significant difference in microplastic morphologies between the seasons, with fragments and films occurring at similarly low frequencies throughout the year. In the summer months, the ratio of fibers to films and fragments was 13:1 and in the winter, it was 14:1.

**Fig. 3:**
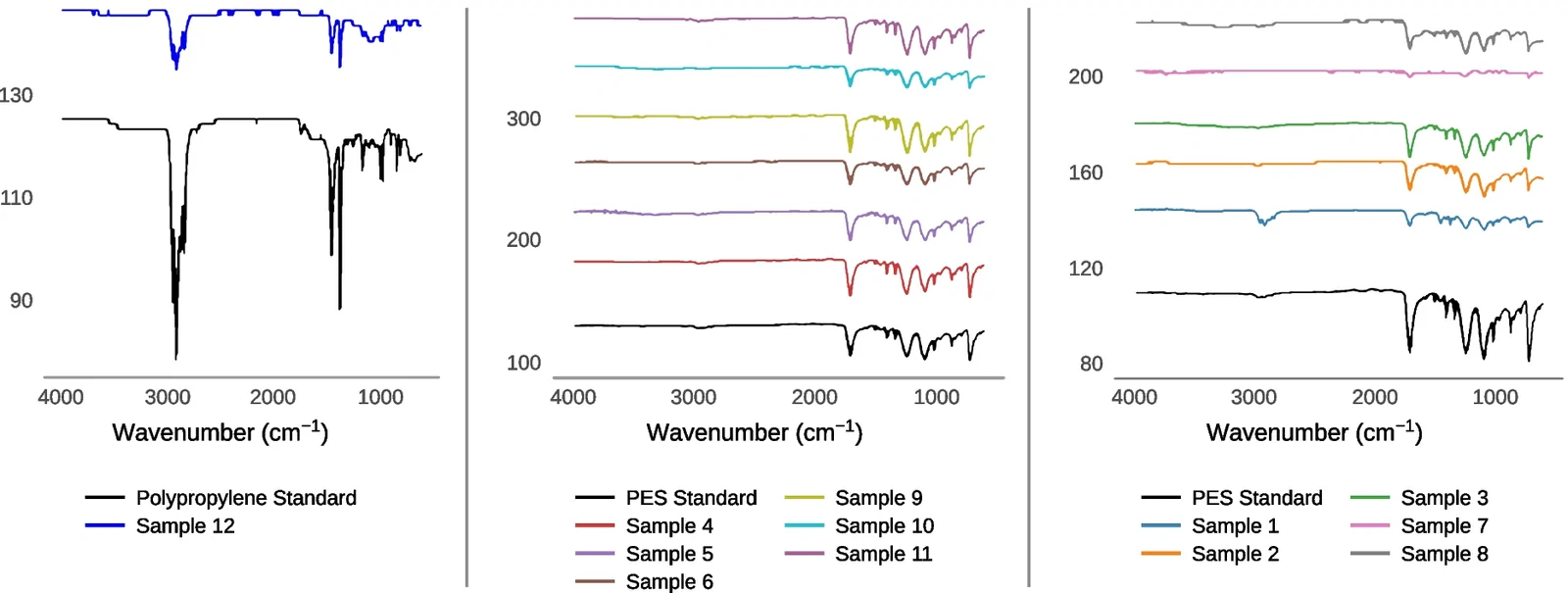
FTIR % transmission spectra of one representative polypropylene sample (left panel, blue) and our polypropylene standard (left panel, black). FTIR transmission spectra of six representative polyester samples with lengths 1–5 mm (center panel, colored), and our polyester standard (center panel, black), and six representative polyester samples with lengths 250 μm–1 mm (right panel, colored), and our polyester standard (right panel, black). Sample 7 (right panel, pink) represents the lowest possible length (250 µm) from which we could generate an identifiable IR spectra. Each of the polyester samples was previously identified as a polyester using the ratio of C:O in the EDS spectra

SEM–EDS analysis of a subset of 75 individual microplastics suggested that 70 (93.3%) were polyester (PES), three (4.0%) were polyvinyl chloride, and two (2.7%) were polyolefins. Among the clusters, five clusters totaling 54 microplastics were found to be polyester (54.5%) and one cluster of 45 fibers was polypropylene (45.5%). Oxygen is known to occur as an artifact in SEM–EDS analyses. To evaluate this contribution, SEM staging discs were analyzed as procedural blanks. Oxygen abundances measured in microplastic particles were consistently far greater than those observed in blank scans, indicating that oxygen associated with plastic spectra reflects sample composition rather than instrumental background contamination (SI Fig. 3). Because SEM–EDS was only a supporting polymer identification technique rather than a definitive one, a subset of 30 microplastics were taken from the SEM–EDS analyses and reanalyzed using FTIR spectroscopy. All fibers analyzed via SEM–EDS that exhibited oxygen peaks in SEM–EDS spectra (*n* = 28) were identified as PES by FTIR analysis, whereas the remaining microplastic particles (*n* = 2), which exhibited spectra dominated by elemental carbon during SEM–EDS analysis, were identified as polypropylene (PP) by FTIR (Fig. 3). Microplastic particles smaller than 250 μm could not be reliably analyzed by FTIR, thereby limiting polymer identification to particles > 250 μm in size. This limitation is evident when FTIR spectra from polyester particles are partitioned by particle size, with particles < 1 mm exhibiting less well-defined transmission peaks relative to particles > 1 mm (Fig. 3).

### Modeling results

The performance of stepwise selection can be compromised by multicollinearity, model overfitting, and final model choice (i.e., selecting one model even though other models may be equally valid) (Hegyi & Laczi, 2015). To address these shortcomings, we eliminated the confounding effects of multicollinearity by removing combinations of model predictors with *R* > 0.8 or VIF values > 10 (SI Fig. 4). These thresholds have been empirically derived (Chan et al., 2022; Kim, 2019; Senaviratna & Cooray, 2019) and, when applied to a stepwise model, reduce the rate of false detection and detection failure (Hegyi & Laczi, 2015). The alias function in R did not identify any perfect collinearity among predictors in our model; however, the vif function found moderate to high levels of multicollinearity between all our interacting variables except for average wind speed × average temperature and discharge × average temperature (SI Fig. 4). All the noninteracting variables were retained.

**Fig. 4:**
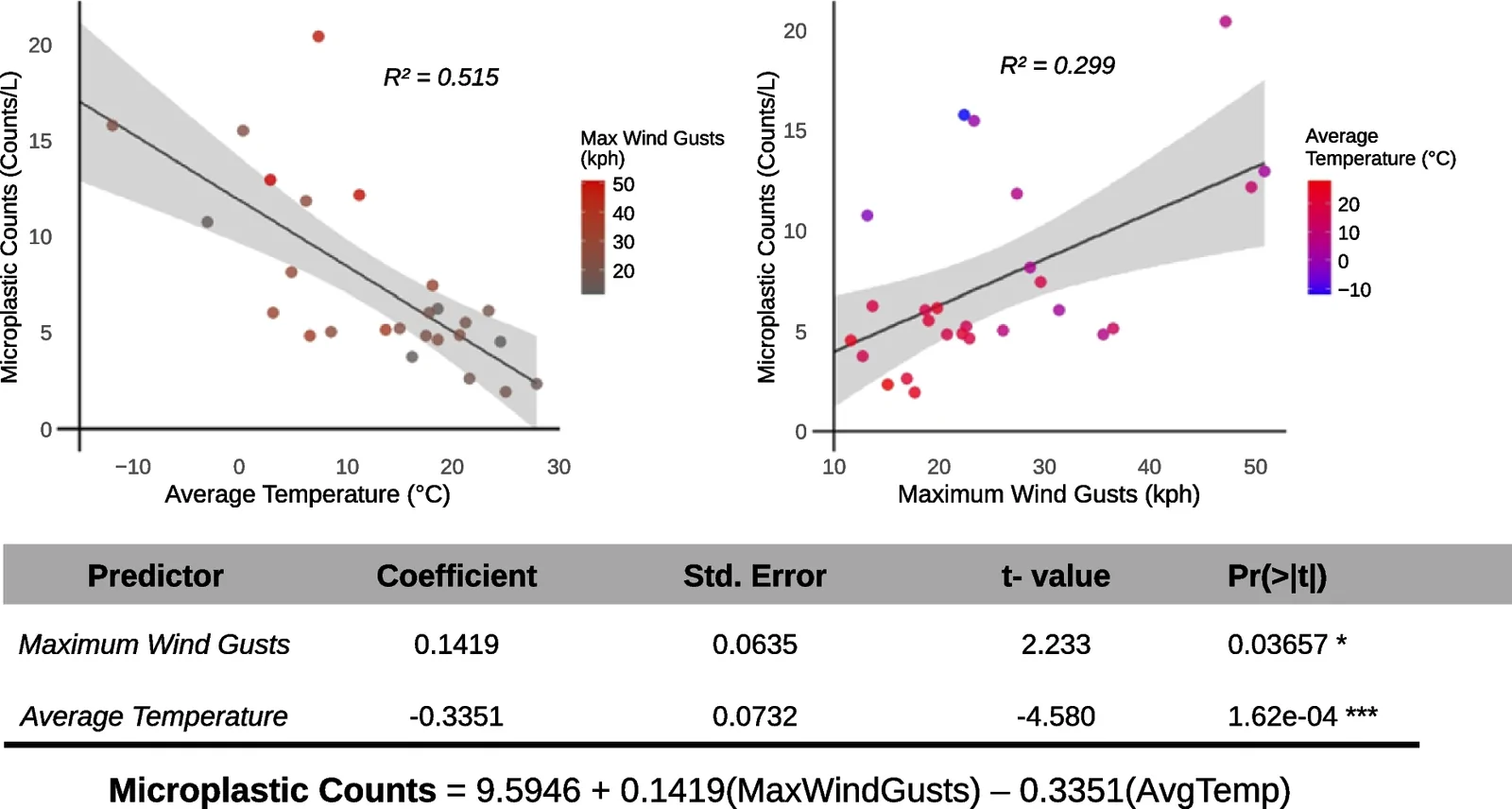
Scatter plots showing the correlation between microplastic counts (particles L^−1^) and temperature (top left) and wind gusts (top right). Our stepwise selection model (bottom table) identified these variables as the two most significant predictors of microplastic concentrations in Plum Run. The equation generated by the stepwise model is displayed at the bottom of the table

The final stepwise selection model identified average temperature and maximum wind gusts as significant predictors of microplastic concentrations in Plum Run (Fig. 4). Specifically, higher wind gusts were positively associated with microplastic counts (*β* = 0.14, *p* = 0.037), whereas higher temperatures were negatively associated (*β* = −0.34, *p* < 0.0002) (Fig. 4). The stepwise model explained approximately 60% of the variation in microplastic concentrations (*R*^2^ = 0.65, adjusted *R*^2^ = 0.60, *F*(4, 21) = 9.78, *p* = 0.0001). Seasonal patterns were also evident, with microplastic concentrations approximately 2.5 times higher from late fall through spring (October–April) relative to the rest of the year. Moreover, concentrations were significantly higher in winter (mean = 11.5, SD = 5.1, *n* = 10) than in summer (mean = 4.7, SD = 1.7, *n* = 12; Welch’s *t* =  − 4.05, df = 10.8, *p* = 0.002), a difference of 6.8 particles L⁻^1^ (95% CI 3.1–10.5).

The LASSO regression model explained a substantial proportion of the variability in microplastic concentrations (*R*^2^ = 0.65; adjusted *R*^2^ = 0.58). Using the λmin penalty parameter, the model retained only average temperature (*β* =  − 0.176) and maximum wind gusts (*β* = 0.038) as predictors, while all other variables were shrunk to zero. The elastic net model produced nearly identical explanatory power (*R*^2^ = 0.65; adjusted *R*^2^ = 0.58) but retained total precipitation, discharge, maximum wind gusts, and average temperature as predictors. Coefficient signs indicated positive relationships with precipitation and wind gusts and negative relationships with discharge and temperature.

Repeated tenfold cross-validation indicated similar predictive performance among all three modeling approaches. The stepwise model achieved a cross-validated *R*^2^ of 0.75 with a RMSE of 3.1 particles L⁻^1^. The LASSO and elastic net models both yielded cross-validated *R*^2^ values of 0.79 and RMSE values of 2.9 particles L⁻^1^. Despite slight differences in predictive performance, all three modeling approaches consistently retained average temperature and maximum wind gusts as important predictors of microplastic concentrations, suggesting that these variables are the strongest and most robust drivers of microplastic abundance in Plum Run (Table 1).

**Table 1:** Cross-validation results of the three linear regression models along with the final predictors equations used in this study to predict microplastic concentrations

Model	*R*^2^	Cross-validated *R*^2^	RMSE
Stepwise	0.79	0.75	3.2
Elastic net	0.80	0.79	2.9
LASSO	0.80	0.79	2.9

## Discussion

### Environmental mechanisms of microplastic delivery

In terms of absolute concentrations, the mean microplastic abundance observed in Plum Run (7.6 particles L⁻^1^; range = 1.2–23.0 particles L⁻^1^) falls within the range reported for other urbanized freshwater systems sampled at relatively fine particle size thresholds. Pham et al. (2024) reported a mean concentration of 9.8 ± 7.9 particles L⁻^1^ in the Jungnang Stream, an urban tributary of the Han River in South Korea, while Witzig et al. (2024) measured concentrations ranging from 1.21 to 13.05 particles L⁻^1^ in a small stream in southern Germany. Similarly, Eo et al. (2019) reported water column concentrations of approximately 0.3–4.8 particles L⁻^1^ in the Nakdong River, South Korea, where microplastics were quantified to a lower size limit of 20 μm. Thus, despite methodological differences among studies, the magnitude and range of water column microplastic concentrations observed in Plum Run are broadly consistent with those reported from other urbanized freshwater systems.

Previous studies have shown that microplastic concentrations in aquatic systems are influenced by seasonal variability; however, the magnitude and environmental mechanisms driving these patterns remain highly variable. Seasonal and short-term differences have been documented across a range of freshwater systems. For example, Witzig et al. (2024) documented a 10.8-fold change in microplastic concentrations over only 8 days in a small German stream, while Eo et al. (2019) found pronounced seasonal differences in the Nakdong River, South Korea, with approximately 71% of the annual microplastic particles transported during the wet season. Studies from monsoonal regions have frequently linked such variability directly to seasonal precipitation. In the Liangfeng River in southern China, abundances of plastics increased during the rainy season (9.80 ± 10.33–41.93 ± 8.13 particles L⁻^1^) relative to the dry season (6.20 ± 0.57–29.13 ± 6.90 particles L⁻^1^), leading the authors to conclude that rainfall facilitates the transfer and resuspension of microplastics into the water column (Xia et al., 2023). Similarly, concentrations in the Jungnang Stream increased with precipitation and discharge (Pham et al., 2024). These interpretations are mirrored by studies from other geographic regions in which microplastic concentrations or transport increased proportionally with discharge and precipitation (Mora-Teddy & Matthaei, 2020; Moses et al., 2023).

However, substantial temporal variability does not necessarily indicate hydrologic control. Despite the more than tenfold variation observed by Witzig et al. (2024), microplastic concentrations showed no distinct relationship with precipitation or discharge. Similarly, Erni-Cassola et al. (2024) found that microplastic concentrations measured over 22 months in the Rhine River were not well explained by river discharge, indicating that temporal variability in water column concentrations may be decoupled from hydrologic forcing. Collectively, these studies indicate that pronounced temporal variability is common in freshwater systems, but the environmental variables governing that variability can differ substantially among watersheds.

In contrast to hydrologically driven seasonal variability reported for many fluvial systems, our modeling results indicate that cold air temperatures coupled with higher wind gusts are correlated with enhanced microplastic concentrations in Plum Run. Moreover, stream discharge and precipitation amount are nonsignificant drivers of microplastic concentrations, suggesting that the dominant environmental controls on water column microplastics vary among catchments and need not be driven by hydrologic forcing. Together, the strong influence of wind speed and temperature, coupled with the lack of a discharge or precipitation signal, is consistent with a hypothesis in which aeolian deposition represents a major pathway of microplastic delivery to Plum Run.

It is therefore not surprising that delivery mechanisms of microplastics into Plum Run differ from those in the aforementioned studies. We suggest that these differences arise from regional scale environmental conditions that exert a first-order influence on microplastic delivery to each site. Indeed, sites unaffected by monsoonal precipitation show significantly higher microplastic counts during periods of lower temperature and higher wind velocities, with no clear relationship to precipitation (Koutnik et al., 2022; Montecinos et al., 2024; Soininen et al., 2024; Wright et al., 2020). Others have more directly tested the link between wind speed and fine particulate matter pollution across the atmospheric boundary layer and found that small fibrous particles, akin to the microplastics found in this study, were more likely to be entrained in the atmosphere at higher wind speeds (Bullard et al., 2021; Yuan et al., 2023). The high aspect ratios and low effective settling velocities of fibrous particles keep them suspended longer than fragments or films of the same mass, making them even more likely to be entrained and transported as wind speeds increase (Rosal, 2021; Tatsii et al., 2023). This behavior may explain why maximum wind gusts, rather than mean wind speeds, were more strongly associated with microplastic concentrations in Plum Run: short-lived gusts may exceed the velocities required to entrain and resuspend fibrous particles that remain stationary under average wind conditions (Bullard et al., 2021). As gusts subside and atmospheric turbulence weakens, these entrained particles can subsequently settle from suspension, producing a coupled cycle of wind-driven entrainment, transport, and deposition (Rao et al., 2024).

The aspect ratios of fibrous microplastic particles measured in this study (mean = 14) are consistent with particles that have enhanced atmospheric transport potential (Preston et al., 2023; Tatsii et al., 2023; Xiao et al., 2023). Model simulations of plastic transport and deposition across a range of particle lengths and aspect ratios found that deposition rates greatly increase among particles with lengths ≥ 125 μm and aspect ratios between 10 and 100 (Ward et al., 2024). Given the similarity of these results to our data, the fibrous particles found within Plum Run exhibit morphologies consistent with enhanced potential for atmospheric suspension and transport.

We propose two hypothesized mechanisms that may help explain the observed association between lower temperatures and elevated microplastic concentrations which are consistent with increased atmospheric deposition: seasonal changes in vegetative interception and cold season increases in atmospheric microplastic emissions and transport. First, from the period of leaf flush to senescence, forested areas likely function as barriers to microplastic deposition in aquatic systems. There is an established link between vegetation cover and particulate matter entrapment (Koutnik et al., 2022). Forested zones in metropolitan areas capture approximately 2.2 billion airborne microplastic particles per year (Sunaga et al., 2024). Epidermal wax in deciduous trees, which can trap airborne microplastic particles (Ullah et al., 2021; Yin et al., 2021), grows at a maximum during the spring and summer seasons and significantly decreases during the autumn and winter (Steinparzer et al., 2023). Microplastics are therefore more likely to accumulate on leaf surfaces during the warmer months because of this enhanced physical trapping mechanism. During colder months, particle transport pathways become less obstructed, effectively increasing the rate of deposition onto terrestrial and aquatic surfaces when foliage coverage decreases. Second, mechanical agitation of synthetic textiles in tumble dryers is known to produce microplastic fibers, and fiber release from this process would be expected to increase as more, and more densely woven, textile fabrics are worn during colder months (Torres-Agullo et al., 2021). We hypothesize that the transport and deposition of these fibers into streams could be amplified as wind velocity increases, potentially facilitating atmospheric transport and deposition onto stream surfaces and adjacent riparian zones (De Falco et al., 2019; Tatsii et al., 2023). Taken together, these mechanisms indicate that lower temperatures and higher wind speeds could enhance microplastic delivery to aquatic systems across temperate latitudes by reducing leaf coverage and increasing the atmospheric transport efficiency of microplastic fibers.

### Atmospheric deposition modeling

To test the plausibility of aeolian microplastic delivery, we regressed wind gust data (miles day^−1^) against plastic flux (particles day^−1^) in Plum Run (*n* = 49, *R*^2^ = 0.42, *p* < 0.001). This creates a first-order environmental model to estimate the amount of daily microplastic discharge statistically associated with wind speed, assuming the regression relationship holds and other processes are captured in the intercept and residuals. Daily microplastic flux increased with average wind speed, explaining a moderate fraction of variance. The mean wind-sensitive component predicted by the model averaged 1.28 × 10^7^ particles day^−1^, which is comparable to the mean observed stream water flux of 1.38 × 10^7^ particles day^−1^, and the model accurately reproduced the mean flux across all samples. The remaining particles likely reflect baseline sources and in-stream or watershed processes not captured by wind delivery alone. Together, these results are consistent with a mechanism in which wind-driven mobilization and deposition in the channel or riparian zone contributes substantially to observed microplastic export, though the regression represents an empirical association rather than a direct mass balance.

If we assume that atmospheric delivery of microplastics to the stream is mostly derived from particles deposited near the stream channel, then we can calculate a plastic deposition rate normalized to the area of the riparian zone using

2 $$D={F}_{\mathrm{w}\mathrm{i}\mathrm{n}\mathrm{d}}/{A}_{\mathrm{r}\mathrm{i}\mathrm{p}}\times \eta$$

where *D* is the deposition rate in particles m⁻^2^ day⁻^1^, *F* is the portion of microplastic export associated with wind derived from our regression model in particles day⁻^1^ (as an annual average), *A* is the area (m^2^) of the riparian buffer zone, and *η* is the fraction of deposited particles that reach the stream and are exported on that day (unitless). We define *A* by the area of the Plum Run riparian zone using

3 $${A}_{\mathrm{r}\mathrm{i}\mathrm{p}}=2\times L\times w$$

where *L* is the length of the stream in meters (19,300 m) and *w* is the width (m) of the riparian buffer. We set *w* to 50 m based on common literature values associated with maximum pollution removal in riparian zones (Mayer et al., 2007). Direct estimates of same-day aeolian delivery ratios for microplastics are not well constrained in the literature; however, catchment-scale *η* values and modeled retention efficiencies indicate that only a fraction of terrestrial microplastics is exported to streams (Schmidt et al., 2017). We selected a delivery ratio of *η* = 0.01 as a conservative estimate of same-day export, consistent with literature showing strong retention of microplastics within terrestrial and fluvial compartments (Sajjad et al., 2022).

The implied atmospheric microplastic deposition rate calculated using Eq. 2 is 663 particles m⁻^2^ day⁻^1^. To evaluate the sensitivity of this estimate to our assumed parameter values (i.e., *η* and *w*), we conducted a sensitivity analysis showing the implied deposition rate *D* and the wind-associated export *F*_wind_ across a range of riparian widths (*w* = 10–150 m) and delivery ratios (*η* = 0.01–0.10) (Supplemental Table 2). Deposition rates span 22–3161 particles m⁻^2^ day⁻^1^ across this parameter space. All atmospheric deposition values, regardless of *η* or *w*, fall within the range of atmospheric microplastic deposition rates previously reported in urban, suburban, or rural environments, where fluxes commonly range from tens to several thousand particles m⁻^2^ day⁻^1^ (Roblin et al., 2020; Sun et al., 2022; Xu et al., 2024; Zhang et al., 2020). The deposition rate we calculated using *w* = 50 m and *η* = 0.01 (663 particles m⁻^2^ day⁻^1^) falls within the lower-to-middle range of these reported depositional rates and was selected based upon the population density of our study area relative to those studies. We therefore consider these parameter values to represent a conservative but physically plausible estimate of atmospheric microplastic delivery to the stream corridor. The consistency between our calculated deposition rate and previously reported atmospheric deposition fluxes supports the physical plausibility of wind-driven atmospheric deposition as a potential source of microplastics to Plum Run without offering a full validation of our proposed mechanism.

### Sources of microplastics

Since the commercialization of polyester and other synthetic textiles in the 1950 s, global per person fiber production has increased by 80% from 1975 to 2022, and polyester production was 63 million tons in 2022 (Textile Exchange, 2024). According to the US Energy Information Administration, in 2020, 68% of Americans used their dryer for 1–4 times a week, while 26% do 5–9 loads per week, equating to approximately 52,468 dryer cycles in a year per housing unit (US EIA, 2020). Machine drying of synthetic clothing triggers chemical and mechanical processes that release polyester fibers that were previously bound to the underlying textile (De Falco et al., 2019). Indeed, a single dryer cycle can release more than 500,000 synthetic microfibers into the environment (Tao et al., 2022).

Characterization of Plum Run microplastics using SEM/EDS consistently identified peaks in the fibrous particles isolated from our water column samples that correspond to molecular oxygen (SI Fig. 3). These results indicate the presence of an ester linkage which forms the basis of polyester plastics by linking hydrocarbon monomers into larger polymer chains. All members of the subset of larger microplastic particles analyzed via SEM–EDS were confirmed to be polyester using ATR-FTIR (Fig. 3). Polyethylene terephthalate (PET) is a thermoplastic polymer, widely used for various packaging, bottles, and textiles (Pinlova & Novack, 2024). Thus, the subset of fibers confirmed as polyester through FTIR could be derived from a range of sources. However, because all our polyester particles were fibers and microplastic fibers are the most common plastic morphotype generated from textiles (Cai et al., 2020a, 2020b), we argue that the PES contributions to our stream system are likely derived from textiles.

Further, rapid increases in microplastics in aquatic systems are linked to higher residential densities (Kunz et al., 2023) and microplastic abundances scale inversely with the distance from residential areas (Kunz et al., 2023). With residential land use covering 50.3% of the Plum Run watershed, we argue that elevated microplastic concentrations during colder months are consistent with a contribution from synthetic textile fibers, as clothes drying and mechanical wear of synthetic garments during colder months represent high-flux fiber sources (Cai et al., 2020b; Torres-Agullo et al., 2021). The magnitude of this pollution is expected to scale with the amount and density of clothing worn, as well as the frequency with which these garments are subsequently washed and dried. Although other potential contributors of polyester fibers may also contribute microplastics to Plum Run (e.g., road dust, wastewater inputs, and other urban sources), the magnitude of synthetic textile production and use is remarkable and provides a plausible source for the observed polyester fibers. Each year 111 million metric tons of clothing fibers are produced for the textile industry (Shirvanimoghaddam et al., 2020), and in the USA, the average person discards about 37 kg of clothing annually (U.S. Environmental Protection Agency, 2021). Since the majority of this clothing is composed of synthetic fibers (Chen et al., 2023) and because heavier clothing is more frequently worn, washed, and dried in colder months, the observed temperature signal is consistent with increased textile fiber emissions during colder months, which could be transported atmospherically and deposited in Plum Run.

### Limitations

This study presents a long-term, twice-monthly sampling study of microplastic pollution in a small Piedmont stream from a predominantly urban watershed. The temporal resolution recorded here demonstrates that higher-resolution sampling is necessary to capture the high variability in microplastic concentrations over annual cycle, thereby providing a substantial improvement over previous studies where results from single or seasonal sampling regimes may not fully capture environmentally driven trends (Kukkola et al. (2024). Our inference of wind-driven atmospheric delivery is based on a statistical association between meteorological variables and stream microplastic concentrations rather than on direct atmospheric deposition measurements. Thus, future studies that model environmental controls on microplastic abundances would benefit from direct measurements to confirm this pathway. Second, since polymer identification via FTIR was limited to particles > 250 μm, our compositional characterization of the smaller size fractions relies on SEM–EDS screening alone. The results from our SEM–EDS analyses were consistent with FTIR polymer assignments across all particles where elemental oxygen was detected by EDS, but since SEM–EDS was only used as a screen tool that cannot definitely identify polymer type, we recommend the use of micro-FTIR to more fully characterize the polymer composition of these smaller compounds.

## Conclusion

Our study examined the relationship between microplastic concentrations in Plum Run and key environmental variables. Linear regression analyses revealed significant associations between microplastic abundances and periods of lower temperature and higher magnitude maximum wind gusts. Notably, all three modeling approaches (Stepwise, LASSO, and elastic net) consistently retained these variables as important predictors of microplastic concentrations. Repeated tenfold cross-validation indicated that the models explained approximately 80% of the variability in microplastic concentrations, underscoring the robustness of temperature and maximum wind gusts as predictors of microplastic abundance in this system. Microplastic compositional analyses conducted using SEM–EDS and FTIR identified a subset (*n* = 28) of our sampled particles as polyester fibers. Coupled with the observed influence of lower temperatures and higher wind gusts on microplastic concentrations in the water column, we suggest that anthropogenic activity is a plausible primary determinant of microplastic abundances. Specifically, increased washing and drying of synthetic textiles during winter months releases polyester fibers into the atmosphere, where they can be transported by wind and subsequently deposited into aquatic systems, consistent with our statistical associations. We find that daily microplastic flux from Plum Run increases with wind speed and that the magnitude of the wind-associated component inferred from this relationship is quantitatively consistent with atmospheric deposition when evaluated over a 50-m riparian contributing area, supporting wind-driven delivery as a meaningful contributor to stream microplastic export. Direct atmospheric measurements using deposition collectors and tracer-recovery experiments would be required to confirm this pathway. This effect may be further amplified by reduced leaf cover during colder seasons, facilitating greater rates of deposition into the stream environment.

Our findings underscore the highly variable nature of microplastic pollution in aquatic systems and emphasize the growing need for long-term, high-resolution sampling within these settings in order to accurately assess and manage plastic pollution and its effects. Additionally, this study highlights that deposition of fibrous microplastic particles is linked to both human influences and environmental factors.

## Supplementary Information

Below is the link to the electronic supplementary material.

ESM 1 (DOCX 2.36 MB)

ESM 2 (XLSX 14.7 KB)

## Data Availability

All the data in this study is made available in the supplementary data file.

## Declarations

All authors have read, understood, and complied as applicable with the statement on “ethical responsibilities of authors” as found in the instructions for authors.
