# Supplementary material for: Seasonality and maximum wind gusts correlate with microplastic concentrations in a freshwater stream system
Source: Environ Monit Assess. 2026 Oct 9;198(11):1173. doi: 10.1007/s10661-026-15985-9 (PMC13652910; doi:10.1007/s10661-026-15985-9)
Supplement: Supplementary file 1 [file 10661_2026_15985_MOESM1_ESM.docx]

| A  0.5 mm | B  0.5 mm |
| --- | --- |
| C  0.5 mm | D  0.5 mm |
| E  1 mm | F  0.5 mm |

**Supplemental Figure 1.** Light microscopy images of microplastic fibers (A-F), as well as their date of collection.

**Supplemental Figure 2.** Plastic standards used in this study (top) alongside the FTIR transmission spectra (middle), and the EDS spectra (bottom) of the same standards. Within each EDS spectra are the characteristic elemental ratios we used to characterize our microplastics via EDS. Standards that only contained carbon and hydrogen were grouped together as polyolefins, as EDS analyses cannot differentiate elemental ratios among standards within these grou ps.

**Supplemental Figure 3.** SEM microplastic images (top panel), representative EDS spectra (middle panel) generated from microplastic samples, and EDS spectra of staging discs (i.e., procedural blanks). The presence of elemental oxygen in these and other samples was used to assign these as polyester polymers. The lack of oxygen spectra in blank scans, or its minimal signal, demonstrates that the measured oxygen is intrinsic to the plastics and not attributable to background or mounting artifacts.

**Supplemental Figure 4.** Pairwise correlation coefficient plot between the environmental variable included in our models. Each cell value reflects the strength and direction of the linear relationship between them.

**Supplemental Table 1.** FTIR characteristic peak assignment for our plastic standards

| **Polymer** | **FTIR Transmission Assignment** | **Characteristic Peaks (cm ^-1^)** |
| --- | --- | --- |
| Polyester (PE) | C = O stretching  C – O stretching  Aromatic CH out-of-plane bending | 1711  1242, 1095  722 |
| High density polyethylene (HDPE) | C-H stretching  CH_2_ bending | 2913, 2847  1471 |
| Polyvinyl chloride (PVC) | CH_2_ bending  CH bending  C – C stretching  CH_2_ rocking  C – Cl stretching | 1424  1331, 1243  1099  965  610 |
| Low density polyethylene (LDPE) | C – H stretching  CH_2_ bending  CH_2_ rocking | 2914, 2848  1471  716 |
| Polypropylene (PP) | C – H stretching  CH_2_ bending  CH_3_ bending  CH bending, CH_3_ rocking, C – C stretching  CH_3_ rocking, CH_3_ bending, CH bending  CH_3_ rocking, C – C stretching  CH_2_ rocking, C- CH_3_ stretching  CH_2_ rocking, C – C stretching, C – CH stretching | 2949, 2916, 2866, 2837  1455  1375  1166  997  972  840  808 |
| Polystyrene (PS) | Aromatic C – H stretching  C – H stretching  Aromatic ring stretching  CH_2_ bending  Aromatic CH bending  Aromatic CH out-of-plane bending | 3024  2847  1601, 1492  1450  1027  695 |
| Nylon (all polyamides) | N – H Stretching  CH stretching  C = O stretching  NH bending, C – N stretching  CH_2_ bending  NH bending, C = O bending | 3291  2930, 2857  1630  1530  1463, 1369  680 |

**Supplemental Table 2.** Sensitivity analysis showing the implied atmospheric microplastic deposition rate (D; particles m⁻² day⁻¹) calculated across a range of assumed riparian buffer widths (w = 10–150 m) and delivery ratios (η = 0.01–0.10). Values highlighted in yellow represent the parameter combination used in this study (w = 50 m; η = 0.01), which yielded an implied atmospheric deposition rate of 663 particles m⁻² day⁻¹.

| **η** | **W (m)** | **D (particles m⁻² day⁻¹)** |
| --- | --- | --- |
| 0.01 | 10 | 3316.06 |
| 0.05 | 10 | 663.21 |
| 0.1 | 10 | 331.61 |
| 0.01 | 20 | 1658.03 |
| 0.05 | 20 | 331.61 |
| 0.1 | 20 | 165.80 |
| 0.01 | 30 | 1105.35 |
| 0.05 | 30 | 221.07 |
| 0.1 | 30 | 110.54 |
| 0.01 | 40 | 829.02 |
| 0.05 | 40 | 165.80 |
| 0.1 | 40 | 82.90 |
| **0.01** | **50** | **663.21** |
| 0.05 | 50 | 132.64 |
| 0.1 | 50 | 66.32 |
| 0.01 | 60 | 552.68 |
| 0.05 | 60 | 110.54 |
| 0.1 | 60 | 55.27 |
| 0.01 | 70 | 473.72 |
| 0.05 | 70 | 94.74 |
| 0.1 | 70 | 47.37 |
| 0.01 | 80 | 414.51 |
| 0.05 | 80 | 82.90 |
| 0.1 | 80 | 41.45 |
| 0.01 | 90 | 368.45 |
| 0.05 | 90 | 73.69 |
| 0.1 | 90 | 36.85 |
| 0.01 | 100 | 331.61 |
| 0.05 | 100 | 66.32 |
| 0.1 | 100 | 33.16 |
| 0.01 | 110 | 301.46 |
| 0.05 | 110 | 60.29 |
| 0.1 | 110 | 30.15 |
| 0.01 | 120 | 276.34 |
| 0.05 | 120 | 55.27 |
| 0.1 | 120 | 27.63 |
| 0.01 | 130 | 255.08 |
| 0.05 | 130 | 51.02 |
| 0.1 | 130 | 25.51 |
| 0.01 | 140 | 236.86 |
| 0.05 | 140 | 47.37 |
| 0.1 | 140 | 23.69 |
| 0.01 | 150 | 221.07 |
| 0.05 | 150 | 44.21 |
| 0.1 | 150 | 22.11 |
